# Supplementary material for: Plasmodium falciparum formins are essential for invasion and sexual stage development
Source: Commun Biol. 2023 Aug 18;6:861. doi: 10.1038/s42003-023-05233-y (PMC10439200; doi:10.1038/s42003-023-05233-y)
Supplement: Supplementary file 2 — Supplemental Information [file 42003_2023_5233_MOESM2_ESM.pdf]

SUPPLEMENTARY INFORMATION

Plasmodium falciparum formins are essential for invasion and sexual stage development

Sophie Collier[1,2], Emma Pietsch[1,2], Madeline Dans[3], Dawson Ling[3], Tatyana A. Tavella[1,2], Sash Lopaticki[5,6], Danushka S. Marapana[5], Mohini A. Shibu[1,2], Dean Andrew[1,2], Snigdha Tiash[1,2], Paul J. McMillan[4], Paul Gilson[3], Leann Tilley[1,2], Matthew W.A Dixon[5,6*]

[1]Department of Biochemistry and Molecular Biology, University of Melbourne, Parkville, Victoria 3010, Australia, [2]Bio21 Molecular Science and Biotechnology Institute, University of Melbourne, Parkville, Victoria 3010, Australia, [3]The Macfarlane Burnet Institute for Medical Research, 85 Commercial Road, Melbourne, Victoria 3004, Australia, [4]Biological Optical Microscopy Platform, University of Melbourne, Parkville, Victoria 3010, Australia. [5]Department of Infectious Diseases, Doherty Institute, University of Melbourne, Parkville, VIC 3010, Australia, [6]Walter and Eliza Hall Institute, 1G Royal Parade, Parkville Victoria 3052, Australia.

This PDF file includes:
Supplementary Table 1
Supplementary Figures 1 to 9

Supplementary Table 1 - Primers use in this study.

Primer name	Sequence 5'-3' (restriction sites in lowercase)
FRM1-HR2-F	gaattcCAGATCCATTGTGTGAAGCAC
FRM1-HR2-R	ggcgccGTTCATTATCTTGTTTCTTGG
FRM1-Int-F	CCTAGTGGATAAACCAGAACAATATG
HA-R	TAGTCCGGGACGTCGTACGG
FRM2-Int-F	GGTATCCTTATTAGAGGATAGTGAAC
GFP85-R	ACCTTCACCCTCTCCACTGAC
PF3D7_0702500 _5'flank F	agctctcgagGTAAATCTGTTTAGTTTAGT
PF3D7_0702500 _5'flank R	agctaagcttCTTTACAAAAATTATTTATT
PF3D7_0702500 _3'flank F	agctgcggccgcGTTGCTTTTGGAACTTGTCC
PF3D7_0702500 _3'flank R	agctgagctcCTTCCAGATAGGTGATTTTC

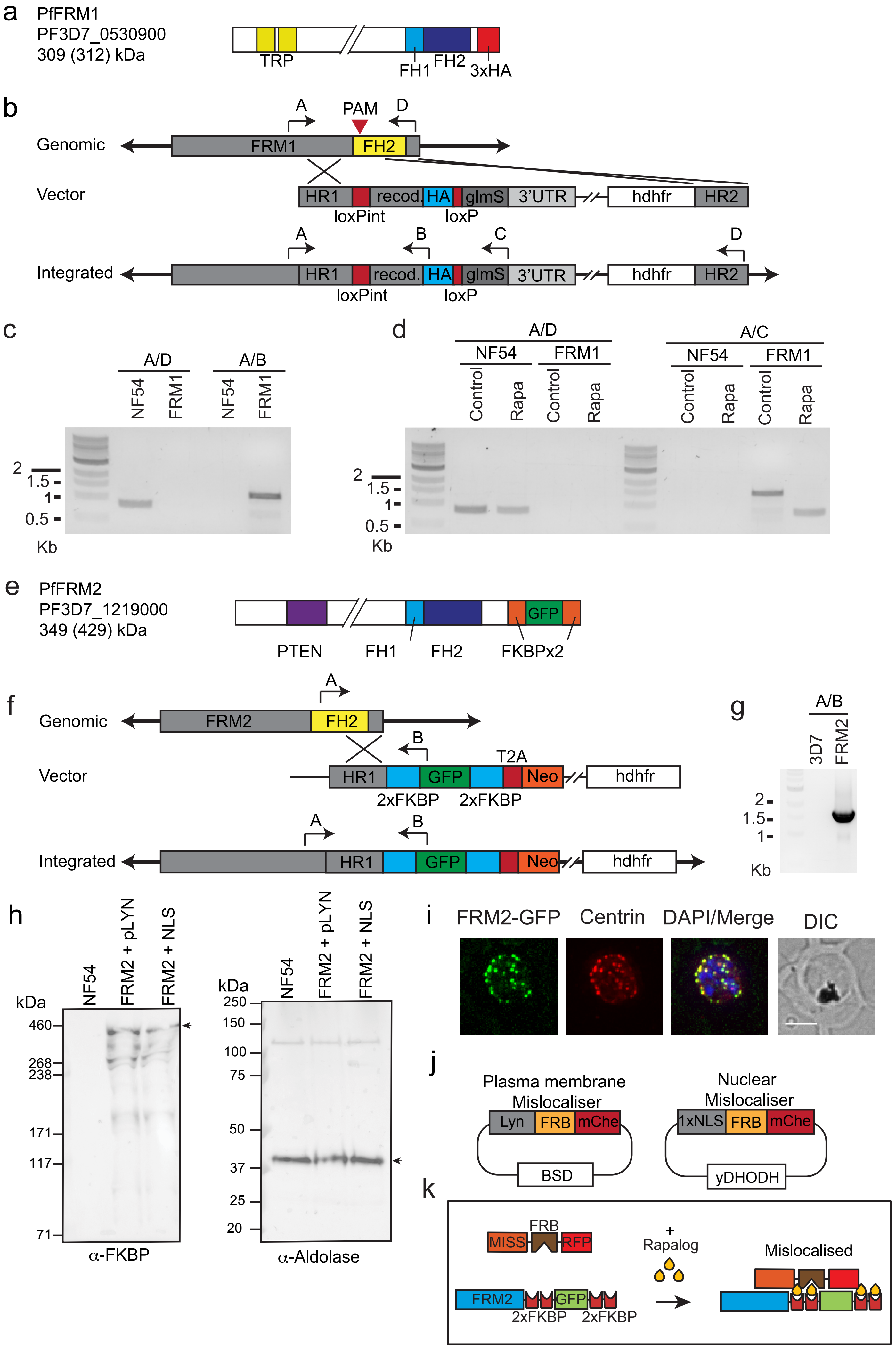
a
PfFRM1
PF3D7_0530900
309 (312) kDa
TRP
FH1
FH2
3xHA
b
Genomic
FRM1
FH2
PAM
A
D
Vector
HR1
recod.
HA
glmS
3'UTR
hdhfr
HR2
loxPint
loxP
Integrated
B
C
c
A/D
A/B
NF54
FRM1
Kb
d
A/C
Control
Rapa
e
PfFRM2
PF3D7_1219000
349 (429) kDa
PTEN
FKBPx2
GFP
f
FRM2
T2A
2xFKBP
Neo
g
3D7
h
FRM2 + pLYN
FRM2 + NLS
kDa
α-FKBP
α-Aldolase
i
FRM2-GFP
Centrin
DAPI/Merge
DIC
j
Plasma membrane Mislocaliser
Lyn
FRB
mChe
BSD
Nuclear Mislocaliser
1xNLS
yDHODH
k
MISS
RFP
+ Rapalog
Mislocalised

Supplementary Fig. 1. Validation of the Formin 1-HA and Formin 2-GFP parasites. a Schematic of Formin 1 (FRM1) protein highlighting the domains and epitope tags used in this study. TRP = tetratricopeptide repeat domain; Formin homology domain 1 and 2 (FH1 and 2); 3xHA = 3x Haemagglutinin tag. Size of protein in kDa (size including tag). **b** Schematic illustrating the genomic locus of FRM1 pre and post integration. HR1/2 = Homology region 1/2; recod. = recodonised 3' FRM1 sequence; loxP sites are shown in red. *glmS* = Glucosamine riboswitch; hdhfr = human dihydrofolate reductase cassette. Arrows with letters indicate the primer positions. **c** PCR validation of pGLMS-FRM1-HA into the genomic locus. Full blots can be seen in Supplementary Fig 9. **d** PCR validation of inducible diCRE gene excision in the NF54-FRM1-HA cell line. Full blots can be seen in Supplementary Fig 9. Wild type NF54 and NF54-FRM1-HA treated with rapamycin (Rapa) or ethanol (Control) are shown. **e** Schematic of Formin 2 (FRM2) highlighting the protein domains and epitope tags used in this study. PTEN = PTEN-C2-like domain; Formin homology domain 1 and 2 (FH1 and 2); GFP = green fluorescent protein; FKBP = FK506-binding protein. Size of protein in kDa (size including tag). **f** Schematic illustrating the genomic locus of FRM2 pre and post integration of the pSLI-FRM2-GFP "FKBP Sandwich" plasmid. FH2 = Formin homology domain 2; GFP = green fluorescent protein; FKBP = FK506-binding protein. T2A = Skip peptide; neo = neomycin cassette. hdhfr = human dihydrofolate reductase cassette. Arrows with letters indicate the primer positions. **g** PCR validation of integration of the pSLI-FRM2-GFP "FKBP Sandwich" plasmid into the FRM2 genomic locus. Full gels can be seen in Supplementary Fig 9. **h** Western blots of lysates from wildtype, pSLI-FRM2-GFP/1XNLS and pSLI-FRM2-GFP/pLYN transfectant parasite lines. Blots were probed with anti-FKBP antibodies to detect the tagged FRM2. Aldolase was used as the loading control. Arrow indicates the full length FRM2 tagged protein and aldolase control. Full blots can be seen in Supplementary Fig. 9. **i** Immunofluorescence microscopy performed on schizont stage FRM2-GFP parasites. Anti-GFP (green) and anti-centrin antibodies (red). DAPI (blue), merge and DIC images are shown. Scale bars = 5 µm. **j** Schematics of the nuclear (1XNLS) and plasma membrane (Lyn) mislocaliser plasmids. FRB = FKBP rapamycin binding protein; mChe = mCherry. BSD = blasticidin cassette; yDHODH = yeast dihydroorotate dehydrogenase. **k** Schematic of the knock-sideways system used to conditionally mislocalise FRM2-GFP to the plasma membrane. In the presence of Rapalog (Rapa), the FKBP and FRB domains dimerise and the protein is mislocalised to the plasma membrane.

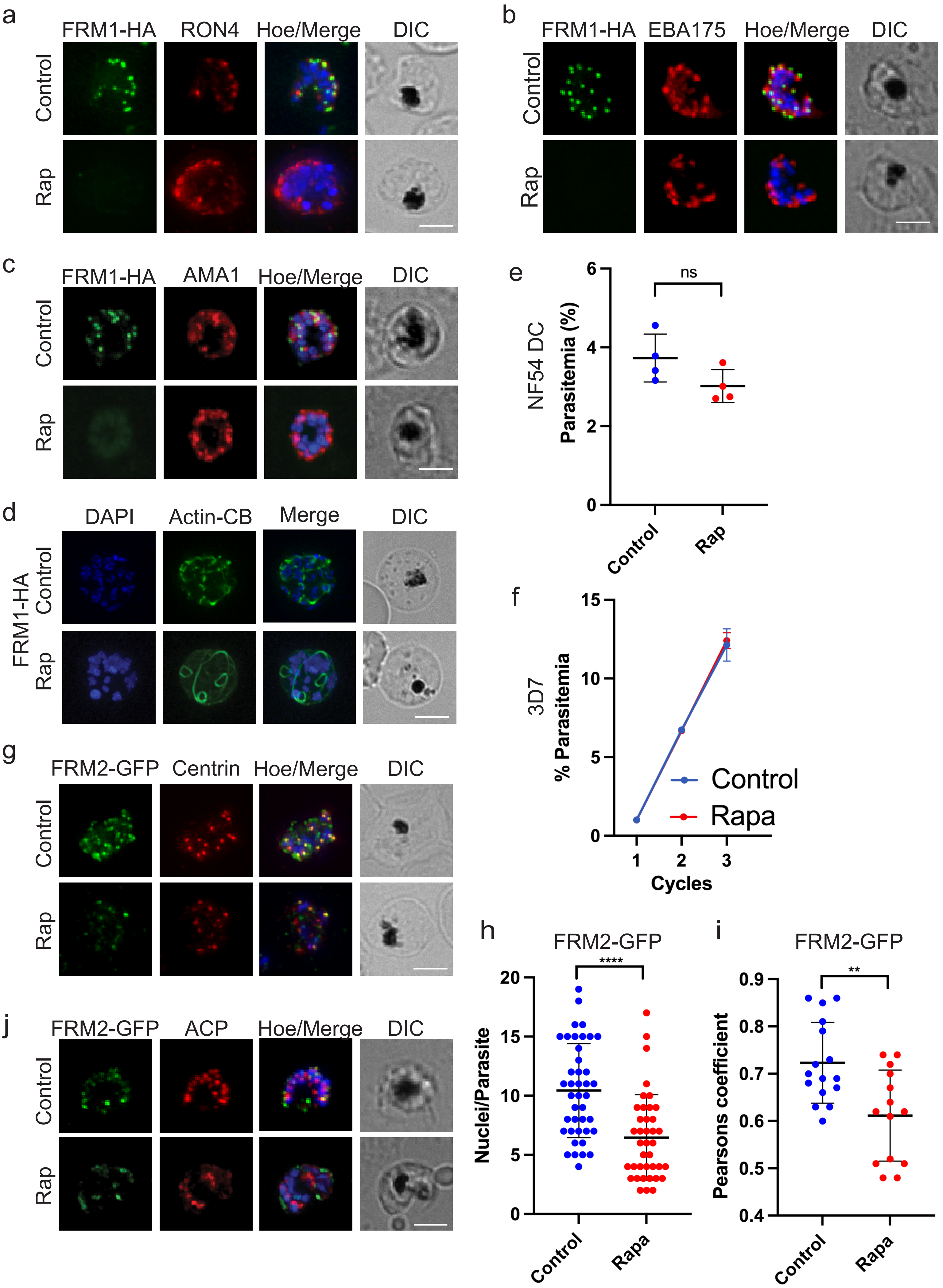

a
FRM1-HA
RON4
Hoe/Merge
DIC
Control
Rap
b
FRM1-HA
EBA175
Hoe/Merge
DIC
Control
Rap
c
FRM1-HA
AMA1
Hoe/Merge
DIC
Control
Rap
d
DAPI
Actin-CB
Merge
DIC
FRM1-HA
Control
Rap
e
NF54 DC
Parasitemia (%)
ns
0
2
4
6
Control
Rap
f
3D7
% Parasitemia
0
5
10
15
1
2
3
Cycles
Control
Rapa
g
FRM2-GFP
Centrin
Hoe/Merge
DIC
Control
Rap
h
FRM2-GFP
Nuclei/Parasite
****
0
5
10
15
20
Control
Rapa
i
FRM2-GFP
Pearsons coefficient
**
0.4
0.5
0.6
0.7
0.8
0.9
Control
Rapa
j
FRM2-GFP
ACP
Hoe/Merge
DIC
Control
Rap

Supplementary Fig. 2. Phenotyping of wildtype controls and FRM1 and 2 transgenic lines. **a-c** Immunofluorescence microscopy of FRM1-HA parasites plus or minus Rapamycin (Rap) labelled with anti-HA (green) and anti-RON4 (**a**), anti-EBA175 (**b**) or anti-AMA1 (**c**, red). Hoechst 33342 (Hoe, blue), merge and DIC images are shown. Scale = 5 µm. **d** Live-cell microscopy of FRM1-HA / Actin chromobody (Actin-CB) schizonts plus or minus Rapamycin (Rap) labelled with Dapi (blue) and Actin-CB (green). Merge and DIC images are shown. Scale = 5 µm. **e** Parasite growth assay NF54 DC (diCRE, FRM1-HA parent line) treated with and without Rapamycin (Rap). Experiments were performed 4 times, overlayed bars represent the mean and standard error of the mean. Differences were not significant. **f** 3D7 parasites (FRM2-GFP parent line). Long term growth assay, parasites were treated from ring stage in cycle 1 and the parasitemia's measured at rings after 1 and 2 cycles. The data is plotted as the accumulative parasitemia over the 3 cycles of growth following Rapalog (Rapa) or ethanol (Control) treatment. Experiments were performed 3 times, the error bars represent the mean and standard error of the mean, differences were not significant. **g** Immunofluorescence microscopy of schizont stage FRM2-GFP parasites following treatment with Rapa or Ethanol (Control). Anti-GFP (green) and anti-centrin (red). Hoechst 33342 (Hoe, blue), merge and DIC images are shown. Scale bars = 5 µm. **h** Quantification of the number of nuclei in Control and Rapa treated schizont stage parasites. Each individual cell count is shown with the mean and standard deviation. Data are from 2 independent experiments. Control n = 41, Rapa n = 39. An unpaired t test was performed. **** = $p < 0.0001$. **i** Analysis of the co-occurrence of centrin and FRM2-GFP. The Pearsons co-efficient was calculated for Control (n= 16 cells) and Rapa (n = 14 cells) treated cells. The experiment was repeated 2 times. The mean and standard deviation is displayed. An unpaired t test was performed. P=0.002 **. **j** Immunofluorescence microscopy of schizont stage FRM2-GFP parasites following treatment with Rapa or Ethanol (Control). Anti-GFP (green) and anti-ACP (red). Hoechst33342 (blue), merge and DIC images are shown. Scale bars = 5 µm. Source data for Supplementary Fig. 2 e, f, h & is provided in the Source Data files.

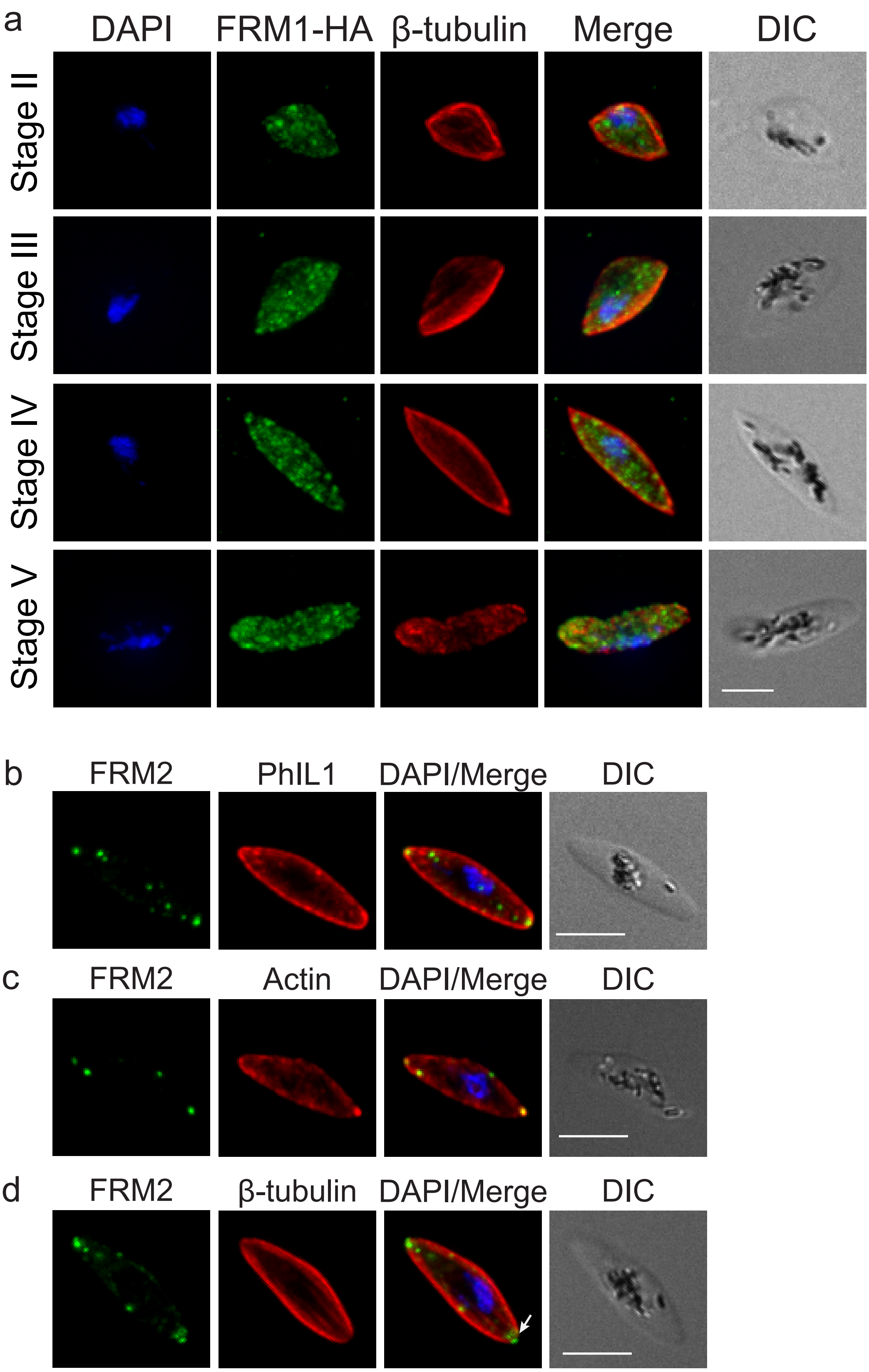

Supplementary Fig. 3. Immunofluorescence assays of FRM1-HA and FRM2-GFP gametocytes.
a Immunofluorescence microscopy of FRM1-HA gametocytes from stage II-V of development with anti HA (green) and anti β-tubulin antibodies (red). DAPI (blue), merge and DIC images are shown. Scale bars = 5 µm. **b-d** Immunofluorescence assays of FRM2-GFP parasites stained with anti-GFP (green) and counterstained with **b** anti-PhIL1; **c** anti-actin; **d** anti-β-tubulin (red). DAPI (blue), merge and DIC images are shown. Scale bars = 5 µm.

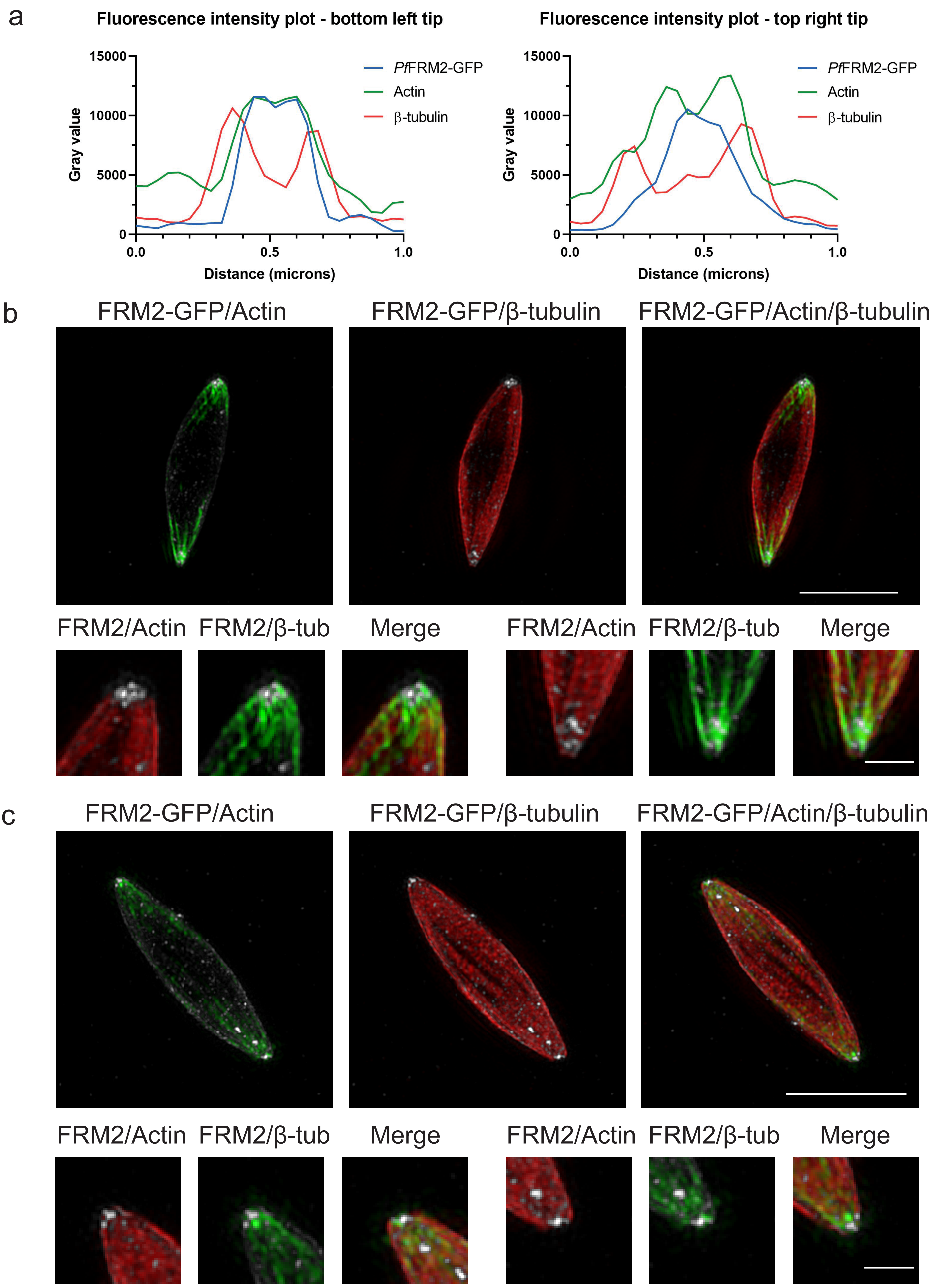

a
Fluorescence intensity plot - bottom left tip
Fluorescence intensity plot - top right tip
Gray value
15000
10000
5000
0
0.0
0.5
1.0
Distance (microns)
PfFRM2-GFP
Actin
β-tubulin
b
FRM2-GFP/Actin
FRM2-GFP/β-tubulin
FRM2-GFP/Actin/β-tubulin
FRM2/Actin
FRM2/β-tub
Merge
c
FRM2-GFP/Actin
FRM2-GFP/β-tubulin
FRM2-GFP/Actin/β-tubulin
FRM2/Actin
FRM2/β-tub
Merge

Supplementary Fig. 4. Additional examples of the 3D-SIM imaging of triple labelled gametocytes. **a** Fluorescence intensity plots of the FRM2-GFP, actin and β-tubulin channels at both gametocyte tips. Red dotted lines in Fig. 3c indicate the lines used for the fluorescence intensity plots of each channel. **b-c** 3D-Structured Illumination microscopy of stage IV gametocytes labelled with anti-GFP (grey), anti-actin (green) and anti-β-tubulin (red). Imaging was performed following actin stabilisation with Jasplakinolide. Merged images of FRM2-GFP/ actin, FRM2-GFP/β-tubulin and all 3 channels are shown. Scale bar = 5 μm. Zoomed images of the top gametocyte tips are shown. Zoom scale bar = 5 μm.

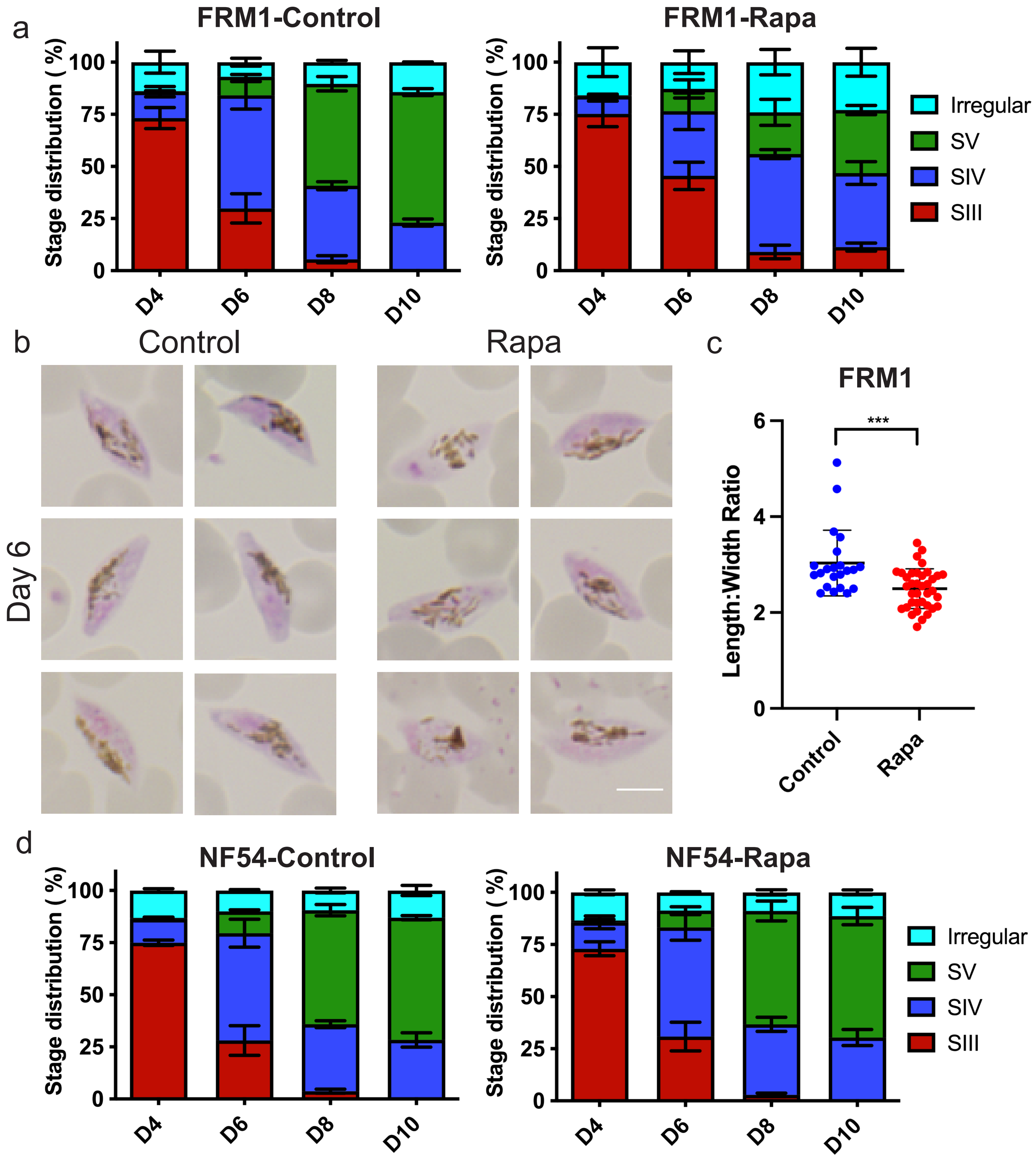

Supplementary Fig. 5. Inducible knockout of FRM1 leads to delayed gametocyte development. **a** Graphs showing the gametocyte stage distribution for FRM1-HA. The relative proportion of each lifecycle stage is shown for Day 4, 6, 8 and 10 of development for control and Rapalog treated parasites. Data from 3 independent experiments each containing 3 repeats are shown. The mean and standard deviation is shown. **b** Representative Giemsa images from day 6. Control (Ethanol) and Rapalog (Rapa) treated parasite images are shown. Scale bar = 5 µm. **c** Length to width ratio of day 6 gametocytes from control and Rapalog treated groups. Control, n =22 and Rapalog, n = 37. The mean and standard deviation is shown. An unpaired t test was performed. p =0.0004 ***. **d** Graphs showing the gametocyte stage distribution for NF54. The relative proportion of each lifecycle stage is shown for day 4, 6, 8 and 10 of development for control and Rapalog treated parasites. Data from 3 independent experiments each containing 3 repeats are shown. The mean and standard error of the mean is shown. Source data for Supplementary Fig. 5a & d is provided in the Source Data files.

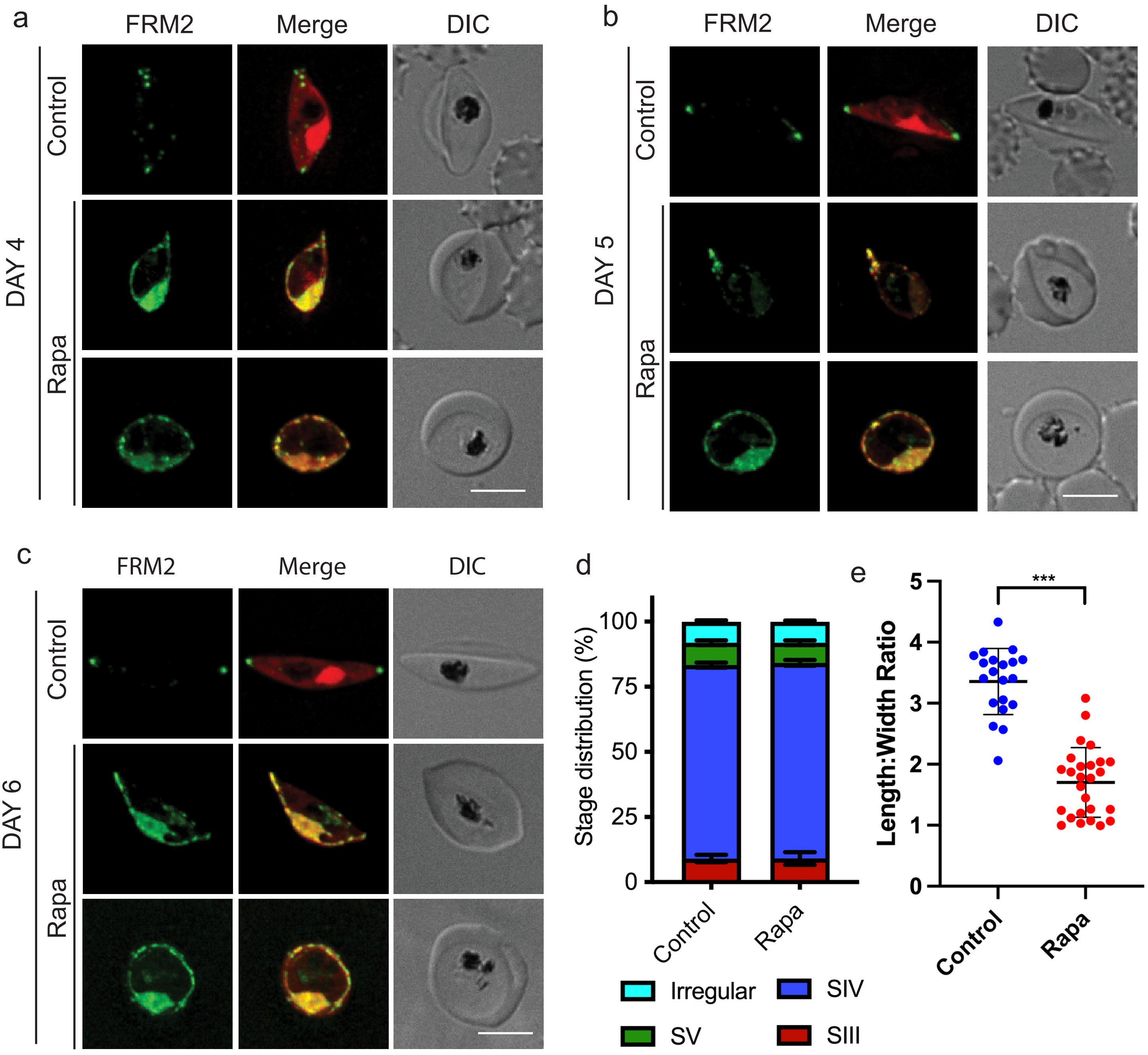

Supplementary Fig. 6. Live cell imaging of FRM2-GFP mislocalization across development. a-c Live cell microscopy of gametocytes from day 4 (**a**), 5 (**b**) and 6 (**c**) of Rapalog treatment. Mislocalisation of FRM2-GFP resulted in both abnormal (middle panel) and rounded morphologies (bottom panel). The nucleus is delineated by the mCherry reporter incorporated into the NLS mislocaliser (red) and FRM2 is labelled with GFP (green). Scale bars = 5 µm. **d** Stage distribution of 3D7-FRM2-GFP parasites without the mislocaliser treated with ethanol (control) or Rapalog continuously for 8 days. The mean and standard error of the mean are shown. Data is representative of 2 experiments each performed in triplicate. **e** Gametocyte length and width measurements following FRM2-GFP mislocalisation. The lengths and widths of live cell images of stage IV gametocytes were measured using ImageJ and the length to width ratio calculated. The mean and standard deviation shown. Control n = 20, Rapa n = 26. An unpaired t test was performed. p = 0.0001 ***. Source data for Supplementary Fig. 6d, e is provided in the Source Data files.

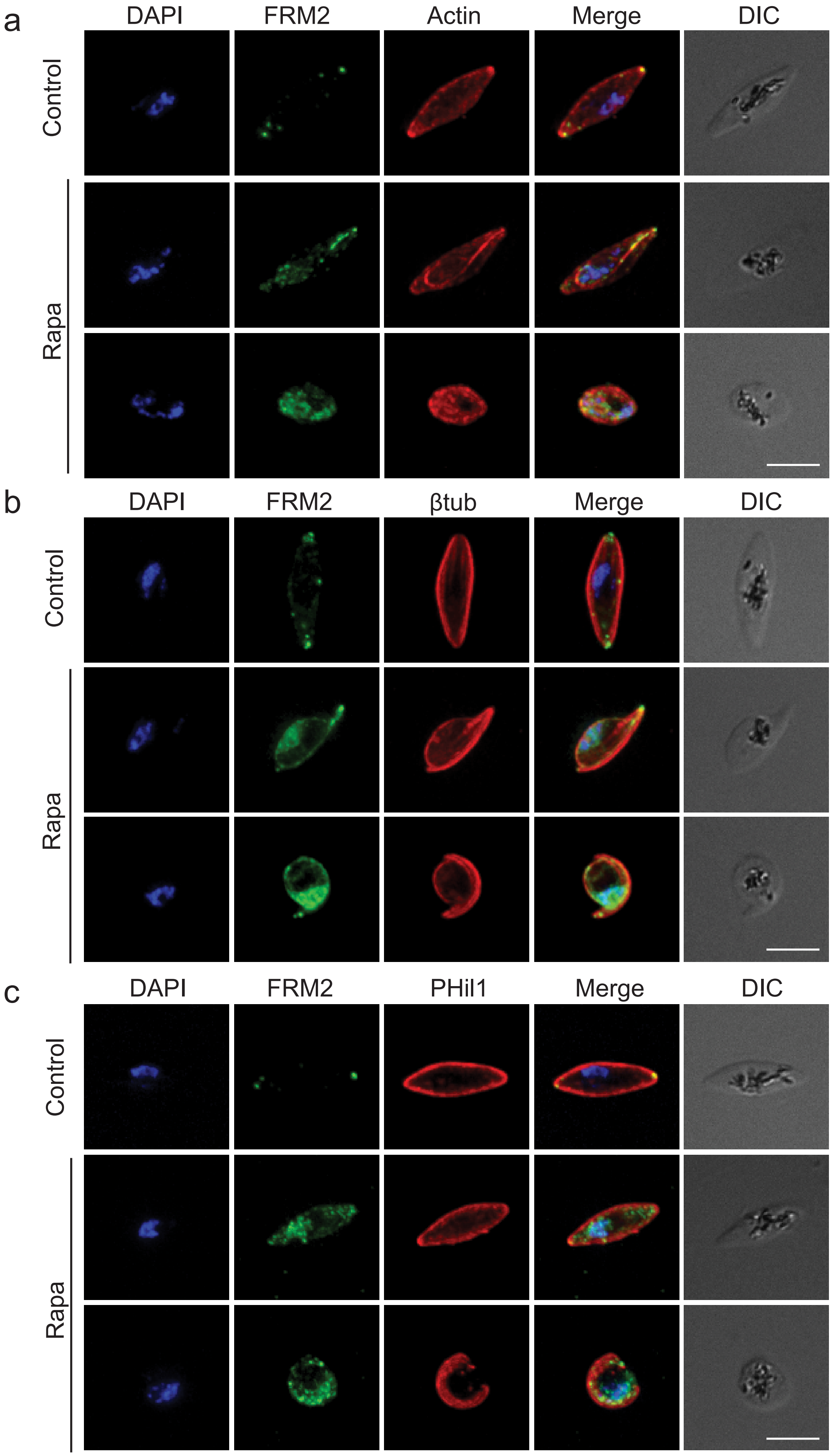

Supplementary Fig. 7. FRM2 disruption affects the cytoskeleton of gametocytes. a-c Immunofluorescence microscopy of Rapalog (Rapa) treated and ethanol treated (control) day 7 gametocytes labelled with anti-GFP (green) and a anti-actin, b anti-β-tubulin or c anti- PhIL1 (red). DAPI (blue), merge and DIC images are shown. Scale bars = 5 μm.

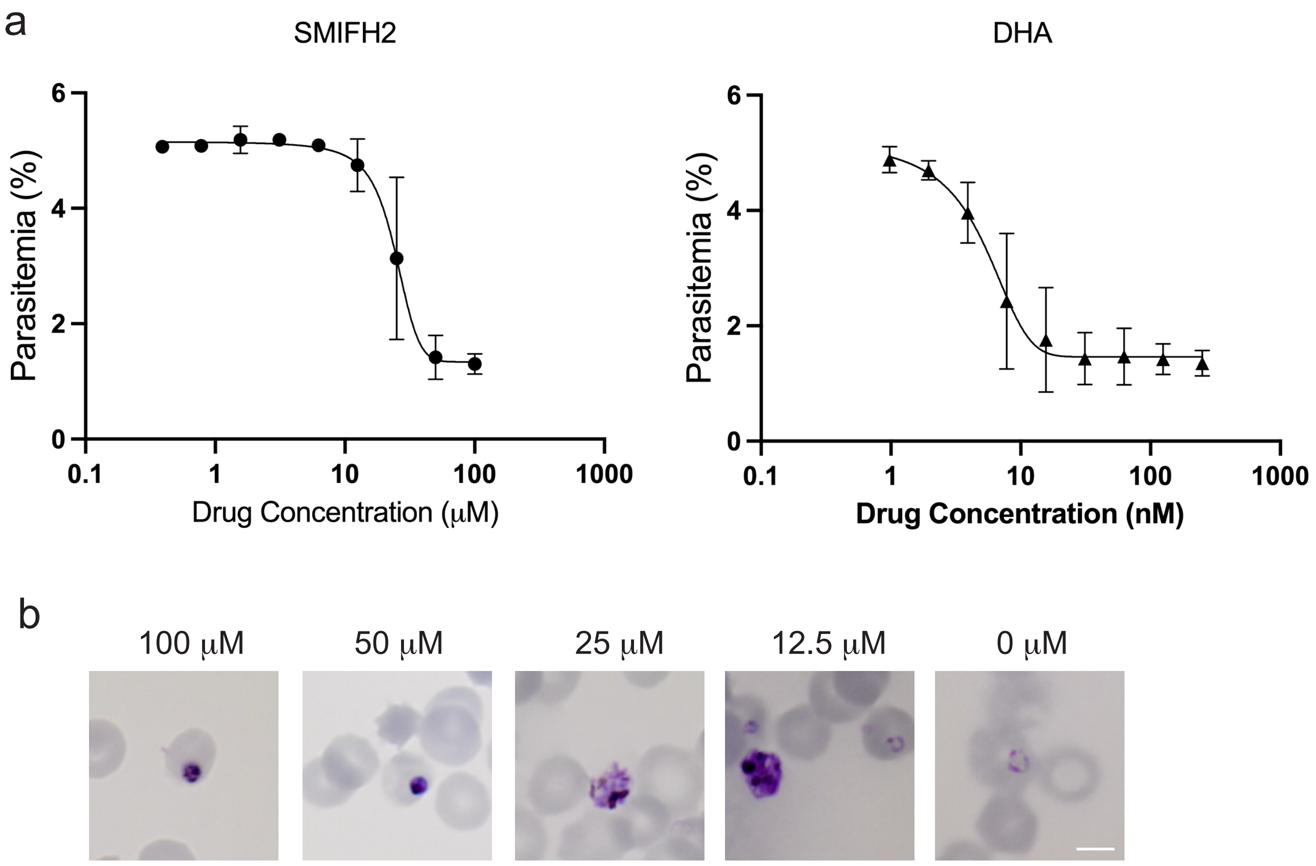

Supplementary Fig. 8. The small molecule inhibitor SMIFH2 kills asexual stage parasites.
a Standard IC50 of asexual stage NF54 parasites. SMIFH2 exhibits an IC50 of 24 ± 6 µM (mean ± SD). Dihydroartemisinin was used as a control. The IC50 value (5 ± 0.6 nM) is similar to literature values. The mean and standard deviation are shown. The experiment was repeated 3 times. **b** Images of asexual parasites after 48h incubation with different concentrations of SMIFH2. Scale bars = 5 µm. Source data for Supplementary Fig. 8a is provided in the Source Data files.

a Supplementary Fig 1c and d

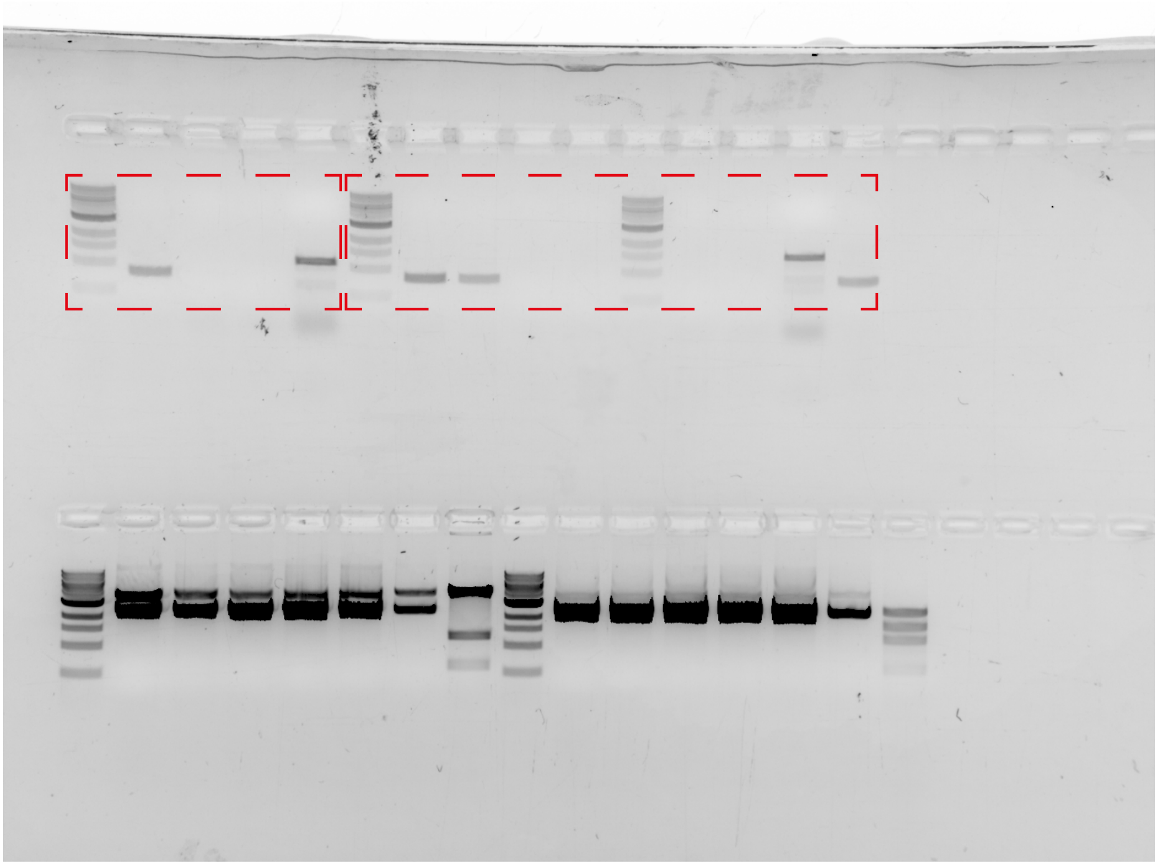

b Supplementary Fig 1g

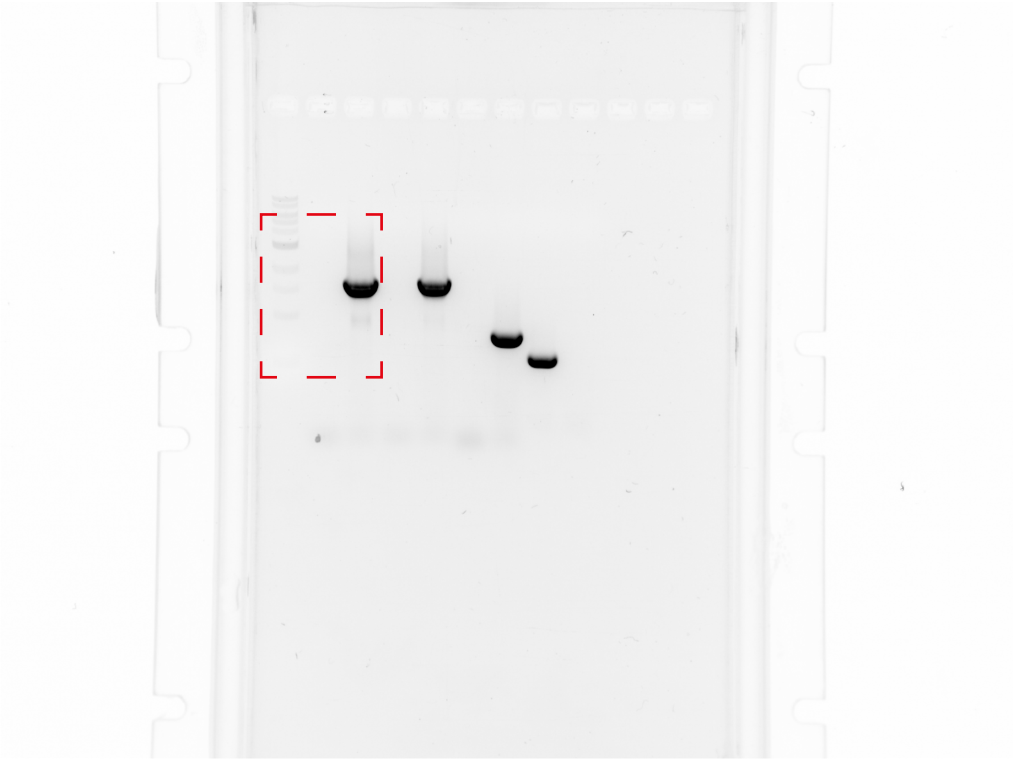

c Supplementary Fig 1h

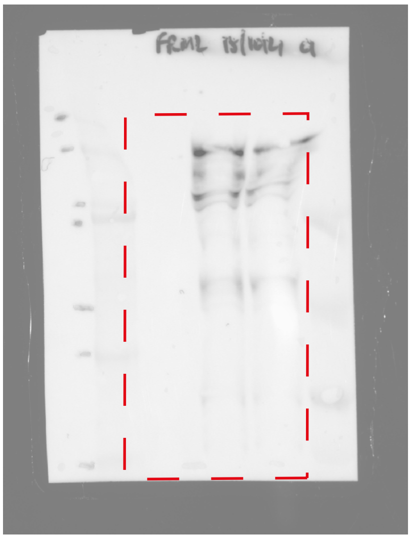

α-FKBP

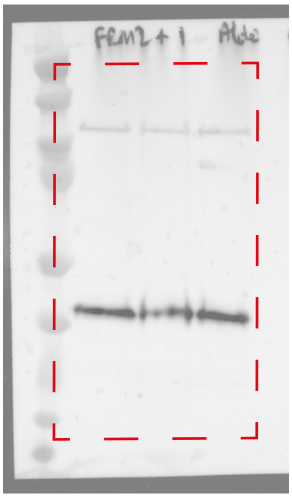

α-Aldolase

Supplementary Fig. 9. Uncropped DNA gels and Western Blots.
a DNA gel from Supplementary Fig 1c and d. **b** DNA gel from Supplementary Fig 1g. **c** Western blots from Supplementary Fig 1h. The red dotted boxes show the regions used in the figures.
