# Supplementary material for: Plasmodium falciparum formins are essential for invasion and sexual stage development
Source: Commun Biol. 2023 Aug 18;6:861. doi: 10.1038/s42003-023-05233-y (PMC10439200; doi:10.1038/s42003-023-05233-y)
Supplement: Supplementary file 3 — Description of Additional Supplementary Files [file 42003_2023_5233_MOESM3_ESM.pdf]

Description of Additional Supplementary Files

File name: Supplementary Video 1
Description: Live cell imaging of a FRM1-HA schizont rupturing and releasing merozoites that invade neighboring RBCs (black arrows).

File name: Supplementary Video 2
Description: Live imaging of an FRM1-HA schizont, pre-treated with Rapalog, rupturing and releasing merozoites that contact but fail to deform and invade RBCs (white arrows). Some merozoites remain tethered to the residual body after schizont rupture (cyan arrows).

File name: Supplementary Video 3
Description: Additional example of live cell imaging of an FRM1-HA schizont, pretreated with Rapalog, rupturing and releasing merozoites that contact but fail to deform and invade RBCs (white arrows). Some merozoites remain tethered to the residual body after schizont rupture (cyan arrows).

File name: Supplementary Data 1
Description: Source Data File – Gametocyte Development Assays

File name: Supplementary Data 2
Description: Source Data File – Asexual Growth Assays

File name: Supplementary Data 3
Description: Source Data File – Image Quantification
