# Supplementary material for: Plasmodium falciparum formins are essential for invasion and sexual stage development
Source: Commun Biol. 2023 Aug 18;6:861. doi: 10.1038/s42003-023-05233-y (PMC10439200; doi:10.1038/s42003-023-05233-y)
Supplement: Supplementary file 10 — Reporting Summary [file 42003_2023_5233_MOESM10_ESM.pdf]

nature portfolio

Corresponding author(s): Matthew Dixon

Last updated by author(s): Aug 4, 2023

Reporting Summary

Nature Portfolio wishes to improve the reproducibility of the work that we publish. This form provides structure for consistency and transparency in reporting. For further information on Nature Portfolio policies, see our Editorial Policies and the Editorial Policy Checklist.

Statistics

For all statistical analyses, confirm that the following items are present in the figure legend, table legend, main text, or Methods section.

n/a	Confirmed	
☐	☒	The exact sample size (*n*) for each experimental group/condition, given as a discrete number and unit of measurement
☐	☒	A statement on whether measurements were taken from distinct samples or whether the same sample was measured repeatedly
☐	☒	The statistical test(s) used AND whether they are one- or two-sided<br>*Only common tests should be described solely by name; describe more complex techniques in the Methods section.*
☒	☐	A description of all covariates tested
☒	☐	A description of any assumptions or corrections, such as tests of normality and adjustment for multiple comparisons
☐	☒	A full description of the statistical parameters including central tendency (e.g. means) or other basic estimates (e.g. regression coefficient) AND variation (e.g. standard deviation) or associated estimates of uncertainty (e.g. confidence intervals)
☐	☒	For null hypothesis testing, the test statistic (e.g. *F*, *t*, *r*) with confidence intervals, effect sizes, degrees of freedom and *P* value noted<br>*Give P values as exact values whenever suitable.*
☒	☐	For Bayesian analysis, information on the choice of priors and Markov chain Monte Carlo settings
☒	☐	For hierarchical and complex designs, identification of the appropriate level for tests and full reporting of outcomes
☒	☐	Estimates of effect sizes (e.g. Cohen's *d*, Pearson's *r*), indicating how they were calculated

Our web collection on statistics for biologists contains articles on many of the points above.

Software and code

Policy information about availability of computer code

Data collection	Restorative wide field deconvolution microscope (DeltaVision DV Elite, Applied Precision); 3D-Structured Illumination Microscope (OMX V4 Blaze, GE Healthcare); Cell observer widefield microscope (Zeiss, AxioCam 702 Mono)
Data analysis	softWoRx 5.0 software (Applied Precision); FIJI(version 2.3.0); GraphPad Prism 8. The following FIJI plugins were used: RGB_Profiler

For manuscripts utilizing custom algorithms or software that are central to the research but not yet described in published literature, software must be made available to editors and reviewers. We strongly encourage code deposition in a community repository (e.g. GitHub). See the Nature Portfolio guidelines for submitting code & software for further information.

Data

Policy information about availability of data

All manuscripts must include a data availability statement. This statement should provide the following information, where applicable:

- Accession codes, unique identifiers, or web links for publicly available datasets
- A description of any restrictions on data availability
- For clinical datasets or third party data, please ensure that the statement adheres to our policy

Additional data are available in Supplementary Information. Source data are provided with this paper. The datasets generated and analysed during the current study are available from the corresponding authors on reasonable request.

Field-specific reporting

Please select the one below that is the best fit for your research. If you are not sure, read the appropriate sections before making your selection.

☒ Life sciences ☐ Behavioural & social sciences ☐ Ecological, evolutionary & environmental sciences

For a reference copy of the document with all sections, see nature.com/documents/nr-reporting-summary-flat.pdf

Life sciences study design

All studies must disclose on these points even when the disclosure is negative.

Sample size

The sample size (n) for each experiment is noted within the respective figure legends (and below).
No sample size calculations were performed.
Fig 1a-c. Images are representative of > 50 cells visualised.
Fig 2a. Images are representative of > 50 cells visualised.
Fig2b. 4 experiments were performed
Fig 2c-f n = 11 (control) and n = 8 (Rapamycin-treated).
Fig 2g. Images are representative of > 50 cells visualised.
Fig 2h. 3 experiments were performed
Fig 3a. Images are representative of > 50 cells visualised.
Fig 3b. Images are representative of > 50 cells visualised.
Fig 3c. n = 15 cells.
Fig 4a. Images are representative of > 50 cells visualised.
Fig 4b. Images are representative of > 200 cells visualised.
Fig 4c. Between 50-100 cells were visualised and their stage determined for each day of development in both treatment groups. 4 experiments were performed
Fig 4d,e. Images are representative of 10 cells visualised in each group and staining combination.
Fig 5a. Images are representative of > 50 cells visualised.
Fig 5b. Between 50-100 cells were visualised and their stage determined for each day of development in both treatment groups. 3 experiments were performed
Fig 5c. Images are representative of > 50 cells visualised.

Supplementary fig 1i. Images are representative of > 50 cells visualised.
Supplementary fig 2a-d. Images are representative of > 50 cells visualised.
Supplementary fig 2e. 4 experiments were performed
Supplementary fig 2f. 3 experiments were performed
Supplementary fig 2g. Images are representative of > 50 cells visualised.
Supplementary fig 2h. Control n =41, Rapa n = 39
Supplementary fig 2i. Control n= 16 cells, Rapa n = 14 cells
Supplementary fig 2j. Images are representative of > 50 cells visualised.
Supplementary fig 3a-d. Images are representative of > 50 cells visualised.
Supplementary fig 4b,c. Images are representative of 10 cells visualised.
Supplementary fig 5 a. Between 50-100 cells were visualised and their stage determined for each day of development in both treatment groups. 3 experiments were performed
Supplementary fig 5b. Images are representative of > 200 cells visualised.
Supplementary fig 5c n = 22 (control), n =37 (Rapa-treated).
Supplementary fig 5 d. Between 50-100 cells were visualised and their stage determined for each day of development in both treatment groups. 3 experiments were performed
Supplementary fig 6a-c. Images are representative of > 50 cells visualised, for each group.
Supplementary fig 6d. Between 50-100 cells were visualised and their stage determined for each day of development in both treatment groups. 3 experiments were performed
Supplementary fig 6e. n Control = 20, RAPA=26
Supplementary fig 7a-c. Images are representative of > 50 cells visualised, for each group.
Supplementary fig 8b . Images are representative of > 200 cells visualised.

Data exclusions

No data was excluded.

Replication

All fluorescence live-cell and immunoflourescence imaging experiments were repeated three times, with comparable results from each experiment.
Live cell invasion assays were performed on four separate occasions.
Live cell analysis of nuclei numbers following knock-sideways of FRM2 was repeated twice.
Gametocyte stage progression assays were all repeated independently three times, with control and rapa-treated groups set up in triplicate.
Asexual stage growth assays were performed in triplicate and on four separate occasions.
All knock-sideways assays were performed in triplicate and on three separate occasions.
SMIFH2 drug assays performed on both asexual and sexual stage parasites were set up in triplicate and performed on three separate occasions.

Randomization

No randomisation was used in this study as morphological identification of the gametocyte stages were required, prior to measurment or

Randomization	analysis. Randomization was not performed for the drug treatment experiments as we assumed the treatment would not alter the data variance.
Blinding	Blinding was used in this study when performing gametocyte stage progression counts and knock-sideways experiment counts. All analysis was performed using semi-automated methods minimizing any biases that may arise. All images within an experiment were acquired using identical microscope settings. All thresholds and analysis metrics were preserved across all measurements.

Reporting for specific materials, systems and methods

We require information from authors about some types of materials, experimental systems and methods used in many studies. Here, indicate whether each material, system or method listed is relevant to your study. If you are not sure if a list item applies to your research, read the appropriate section before selecting a response.

Materials & experimental systems

n/a	Involved in the study	
☐	☒	Antibodies
☐	☒	Eukaryotic cell lines
☒	☐	Palaeontology and archaeology
☒	☐	Animals and other organisms
☒	☐	Human research participants
☒	☐	Clinical data
☒	☐	Dual use research of concern

Methods

n/a	Involved in the study	
☒	☐	ChIP-seq
☐	☒	Flow cytometry
☒	☐	MRI-based neuroimaging

Antibodies

Antibodies used

Western blotting
Primary antibodies:
rabbit anti-FKBP (abcam, Ab24373, 1:2000); rabbit anti-Aldolase (1:1000)
Secondary antibodies:
rabbit HRP-conjugated secondary antibody (Merck, 1:4000); rabbit HRP-conjugated secondary antibody (Merck, 1:4000)

Immunofluorescence microscopy
Primary antibodies:
rabbit anti-actin1.1 (1:300, 21), mouse anti-β-tubulin (clone TUB 2.1, 1:300, Sigma Aldrich), mouse anti-GFP (clones 7.1 and 13.1, 1:300, Roche), mouse anti-centrin (1:100, clone 20H5 mouse, Millipore), rabbit anti-GFP (1:300, rabbit anti-PhIL1 (1:300, chicken anti-GFP (1:500, Invitrogen), mouse anti-HA (clone HA-7, 1:500, Sigma Aldrich), mouse anti-HA-Biotin (clone HA-7, 1:500, Sigma Aldrich), rabbit anti-AMA1 (1:250, EF24, Robin Anders), rabbit anti-EBA175 (1:500, Ab 1552), rabbit anti-ACP (1:500).

Secondary antibodies:
anti-mouse and anti-rabbit IgG-conjugated Alexa Fluor 488, 568 and 647; anti-chicken IgY Alexa Fluor 647; Streptavidin-Alexa Fluor 488 (Life Technologies).

Validation

Western blotting primary antibody:
- rabbit anti-FKBP was used to detect the FKBP domains present in the pSLI-FRM2-GFP "FKBP Sandwich" plasmid. Protein bands of the correct molecular mass were detected, with minimal background and non-specific labeling. Previopusly used in Birnbaum, J., etal. A genetic system to study Plasmodium falciparum protein function. Nature Methods 14, 4, 450-456.
- Aldolase was used as the loading control and has been used previously and validated by Baum, J, et al. A conserved molecular motor drives cell invasion and gliding motility across malaria life cycle stages and other Apicomplexan parasites. J Biol Chem 281, 5197-5208 (2006).

Immunofluorescence microscopy primary antibody:
- mouse anti-centrin was previously validated as a marker of the microtubule organising centre in Plasmodium in Bertiaux E, et al. Expansion microscopy provides new insights into the cytoskeleton of malaria parasites including the conservation of a conoid. PLoS biology 19, e3001020 (2021).
- Rabbit Anti-Phil1 was characterised in Parkyn Schneider, M. et al. Disrupting assembly of the inner membrane complex blocks Plasmodium falciparum sexual stage development. PLoS Pathog 13, e1006659 (2017).
- Chicken anti-GFP was used and validated in this work producing equivalent labeling patterns to endogenous PfFRM2 and immunofluorescnece microscopy performed with the anti-mouse and anti-rabbit GFP antibodies.
- Mouse anti-HA and mouse anti-HA-Biotin have been validated for specificity and cross reactivity by the manufacturer.
- Mouse anti-β-tubulin and rabbit anti-actin1.1 has been previously used and validated in Dearnley MK, et al. Origin, composition, organization and function of the inner membrane complex of Plasmodium falciparum gametocytes. J Cell Sci 125, 2053-2063 (2012).
- Rabbit AMA 1 (Refolded EF24) Kind gift from Robin Anders, Emeritus, La Trobe University.
- Rabbit EBA175 was characterized in Reed, M. B. et al. Targeted disruption of an erythrocyte binding antigen in Plasmodium falciparum is associated with a switch toward a sialic acid-independent pathway of invasion. Proc Natl Acad Sci U S A 97, 7509-7514 (2000).
- Rabbit anti-ACP was characterized in Tonkin, C. J. et al. Localization of organellar proteins in Plasmodium falciparum using a novel set of transfection vectors and a new immunofluorescence fixation method. Mol. Biochem. Parasitol. 137, 13-21 (2004).

All secondary antibodies have been validated for specificity and cross reactivity by the manufacturer.

Eukaryotic cell lines

Policy information about cell lines

Cell line source(s)	Transfected malaria parasite lines based on 3D7 strain (Lawrence G, et al. Effect of vaccination with 3 recombinant asexual-stage malaria antigens on initial growth rates of Plasmodium falciparum in non-immune volunteers. Vaccine 18, 1925-1931 (2000)).<br>Transfected malaria parasite lines based on NF54 (Rh3) DiCre strain. (Wilde, M. L. et al. Protein Kinase A Is Essential for Invasion of Plasmodium falciparum into Human Erythrocytes. mBio 10 (2019)).<br>Transfected malaria parasite lines based on NF54 (Pfs47) DiCre strain. (Knuepfer, E., Napiorkowska, M., van Ooij, C. & Holder, A. A. Generating conditional gene knockouts in Plasmodium - a toolkit to produce stable DiCre recombinase-expressing parasite lines using CRISPR/Cas9. Scientific reports 7, 3881 (2017)).
Authentication	Transfectants validated using PCR with diagnostic primers and Western blot.
Mycoplasma contamination	Cultures tested and shown to be mycoplasma-free.
Commonly misidentified lines (See ICLAC register)	No commonly misidentified lines were used.

Palaeontology and Archaeology

Specimen provenance	Not Applicable
Specimen deposition	Not Applicable
Dating methods	Not Applicable

☐ Tick this box to confirm that the raw and calibrated dates are available in the paper or in Supplementary Information.

Ethics oversight	Not Applicable

Note that full information on the approval of the study protocol must also be provided in the manuscript.

Animals and other organisms

Policy information about studies involving animals; ARRIVE guidelines recommended for reporting animal research

Laboratory animals	Not Applicable
Wild animals	Not Applicable
Field-collected samples	Not Applicable
Ethics oversight	Not Applicable

Note that full information on the approval of the study protocol must also be provided in the manuscript.

Human research participants

Policy information about studies involving human research participants

Population characteristics	Not Applicable
Recruitment	Not Applicable
Ethics oversight	Not Applicable

Note that full information on the approval of the study protocol must also be provided in the manuscript.

Clinical data

Policy information about clinical studies

All manuscripts should comply with the ICMJE guidelines for publication of clinical research and a completed CONSORT checklist must be included with all submissions.

Clinical trial registration	Not Applicable
Study protocol	Not Applicable

Data collection	Not Applicable
Outcomes	Not Applicable

Dual use research of concern

Policy information about dual use research of concern

Hazards

Could the accidental, deliberate or reckless misuse of agents or technologies generated in the work, or the application of information presented in the manuscript, pose a threat to:

No	Yes	
☒	☐	Public health
☒	☐	National security
☒	☐	Crops and/or livestock
☒	☐	Ecosystems
☒	☐	Any other significant area

Experiments of concern

Does the work involve any of these experiments of concern:

No	Yes	
☒	☐	Demonstrate how to render a vaccine ineffective
☒	☐	Confer resistance to therapeutically useful antibiotics or antiviral agents
☒	☐	Enhance the virulence of a pathogen or render a nonpathogen virulent
☒	☐	Increase transmissibility of a pathogen
☒	☐	Alter the host range of a pathogen
☒	☐	Enable evasion of diagnostic/detection modalities
☒	☐	Enable the weaponization of a biological agent or toxin
☒	☐	Any other potentially harmful combination of experiments and agents

ChIP-seq

Data deposition

☐ Confirm that both raw and final processed data have been deposited in a public database such as GEO.

☐ Confirm that you have deposited or provided access to graph files (e.g. BED files) for the called peaks.

Data access links<br>*May remain private before publication.*	Not Applicable
Files in database submission	Not Applicable
Genome browser session<br>(e.g. UCSC)	Not Applicable

Methodology

Replicates	Not Applicable
Sequencing depth	Not Applicable
Antibodies	Not Applicable
Peak calling parameters	Not Applicable
Data quality	Not Applicable
Software	Not Applicable

Flow Cytometry

Plots

Confirm that:

☒ The axis labels state the marker and fluorochrome used (e.g. CD4-FITC).

☒ The axis scales are clearly visible. Include numbers along axes only for bottom left plot of group (a 'group' is an analysis of identical markers).

☒ All plots are contour plots with outliers or pseudocolor plots.

☒ A numerical value for number of cells or percentage (with statistics) is provided.

Methodology

Sample preparation	Asexual stage parasites were set up in triplicate and stained with Syto61 as previously described by Klonis, N. et al. (Artemisinin activity against Plasmodium falciparum requires hemoglobin uptake and digestion. Proc Natl Acad Sci U S A 108, 11405-11410 (2011)). Following staining, cells were analysed by flow cytometry.
Instrument	BD FACSCanto II flow cytometer
Software	BD FACSDiva™ software, data collected was further analysed and plotted using GraphPad PRISM 8
Cell population abundance	500,000 events measured
Gating strategy	Gating is set up to separate stained and unstained cell populations. Infected red blood cells containing live parasites are stained by Syto61 (a red fluorescent stain that binds to nucleic acids), whereas uninfected red blood cells or dead parasites remain unstained.

☐ Tick this box to confirm that a figure exemplifying the gating strategy is provided in the Supplementary Information.

Magnetic resonance imaging

Experimental design

Design type	Not Applicable
Design specifications	Not Applicable
Behavioral performance measures	Not Applicable

Acquisition

Imaging type(s)	Not Applicable
Field strength	Not Applicable
Sequence & imaging parameters	Not Applicable
Area of acquisition	Not Applicable

Diffusion MRI ☐ Used ☐ Not used

Preprocessing

Preprocessing software	Not Applicable
Normalization	Not Applicable
Normalization template	Not Applicable
Noise and artifact removal	Not Applicable
Volume censoring	Not Applicable

Statistical modeling & inference

Model type and settings	Not Applicable
Effect(s) tested	Not Applicable

Specify type of analysis: ☐ Whole brain ☐ ROI-based ☐ Both

Statistic type for inference (See Eklund et al. 2016)	Not Applicable
Correction	Not Applicable

Models & analysis

n/a	Involved in the study
☐	☐ Functional and/or effective connectivity
☐	☐ Graph analysis
☐	☐ Multivariate modeling or predictive analysis

Functional and/or effective connectivity	Not Applicable
Graph analysis	Not Applicable
Multivariate modeling and predictive analysis	Not Applicable
